# Trends in Sexually Transmitted Infections in United States Ambulatory Care Clinics from 2005–2016

**DOI:** 10.3390/jcm11010071

**Published:** 2021-12-24

**Authors:** Ikenna Unigwe, Seonkyeong Yang, Hyun Jin Song, Wei-Hsuan Lo-Ciganic, Juan Hincapie-Castillo, Robert L. Cook, Haesuk Park

**Affiliations:** 1 Pharmaceutical Outcomes and Policy, College of Pharmacy, University of Florida, Gainesville, FL 32610, USA; i.unigwe@ufl.edu (I.U.); yang.se@ufl.edu (S.Y.); hyunjin.song@cop.ufl.edu (H.J.S.); wlociganic@cop.ufl.edu (W.-H.L.-C.); jhincapie-castillo@unc.edu (J.H.-C.); 2 Department of Epidemiology, College of Public Health, University of North Carolina, Chapel Hill, NC 27599, USA; 3 Department of Medicine, University of Florida, Gainesville, FL 32610, USA; cookrl@ufl.edu

**Keywords:** sexually transmitted infection, ambulatory care, HIV, public health, risk factors

## Abstract

We examined the prevalence trends of non-human immunodeficiency virus (HIV) sexually transmitted infections (STI) and associated patient characteristics in U.S. ambulatory-care settings from 2005–2016. We conducted a retrospective repeated cross-sectional analysis using data from the National Ambulatory Medical Care Survey (NAMCS) for individuals aged 15–64 with a non-HIV STI-related visit. Data were combined into three periods (2005–2008, 2009–2012, and 2013–2016) to obtain reliable estimates. Logistic regression was used for analysis. A total of 19.5 million weighted, non-HIV STI-related ambulatory visits from 2005–2016 were identified. STI-related visits per 100,000 ambulatory care visits increased significantly over the study period: 206 (95% CI = 153–259), 343 (95% CI = 279–407), and 361 (95% CI = 277–446) in 2005–2008, 2009–2012, and 2013–2016, respectively (P_trend_ = 0.003). These increases were mainly driven by increases in HPV-related visits (56 to 163 per 100,000 visits) from 2005–2008 to 2009–2012, followed by syphilis- or gonorrhea-related visits (30 to 67 per 100,000 visits) from 2009–2012 to 2013–2016. Higher odds of having STI-related visit were associated with younger age (aged 15–24: aOR = 4.45; 95% CI = 3.19–6.20 and aged 25–44: aOR = 3.59; 95% CI = 2.71–4.77) vs. 45–64-year-olds, Black race (aOR = 2.41; 95% CI = 1.78–3.25) vs. White, and HIV diagnosis (aOR = 10.60; 95% CI = 5.50–20.27) vs. no HIV diagnosis. STI-related office visits increased by over 75% from 2005–2016, and were largely driven by HPV-related STIs and syphilis- or gonorrhea-related STIs.

## 1. Introduction

In the United States (U.S.), it is estimated that over 26 million sexually transmitted infections (STI) were acquired in 2018 [1]. STIs are a major public health concern and burden to the healthcare system. Approximately 66% of STIs are asymptomatic [2] and remain untreated; this increases the risk of acquiring human immunodeficiency virus (HIV), stillbirths, infant death, and infertility [3]. More specifically, syphilis can lead to neurosyphilis, which is a bacterial infection of the brain and spinal cord [3]. Human papillomavirus (HPV) can cause cancer of the cervix, vagina, penis, and anus [4]. Gonorrhea and chlamydia are both causes of pelvic inflammatory disease (PID), which can lead to ectopic pregnancy, tubal infertility, and chronic pelvic pain [3]. A recent study also estimated that STIs account for an annual direct medical cost of USD 16 billion in the U.S. [5]. In recent years, pharmacological products such as the human papillomavirus (HPV) vaccine (first approved in 2006) and pre-exposure prophylaxis (PrEP) (approved in 2012) have received much attention as the concern that vaccination against HPV and the use of PrEP could promote condomless sex by lowering perceived risks of acquiring an STI (risk compensation) [6,7].

Some studies have estimated STI prevalence over the years. However, some of the data used included self-reported STI diagnoses [1,8], which have recall bias. Moreover, due to the sensitivity associated with STIs, there may be reluctance by the patient to divulge such information. Furthermore, the Centers for Disease Control and Prevention (CDC) only requires four STIs (chlamydia, gonorrhea, syphilis, and chancroid) to be reported to the National Notifiable Diseases Surveillance System (NNDSS) [9], creating a need for information on STIs that are not included on that list. These limitations would be minimized if the underlying data were obtained directly from physician offices and included STIs that are not nationally notifiable.

Hence, this study examined prevalence trends of non-HIV STI-related physician office visits (ambulatory care visits that included a diagnosis of an STI other than HIV) and associated patient characteristics using physician-reported, nationally representative data in U.S. ambulatory care settings from 2005–2016.

## 2. Materials and Methods

### 2.1. Study Design and Data

A repeated retrospective, cross-sectional analysis of individuals aged 15–64 years with a non-HIV STI diagnosis during ambulatory care visits from 2005–2016 was conducted, using the National Ambulatory Medical Care Survey (NAMCS) data. NAMCS is a weighted, nationally representative database provided by the CDC of the use of U.S. ambulatory medical care services [10]. NAMCS is based on a national sample of visits to non-federally employed office-based physicians. To ensure accuracy, the SAS codes algorithm provided by the National Center for Health Statistics (NCHS) was used for extraction [11]. The data were reviewed by two authors before and after analysis. This study was exempted by the University of Florida Institutional Review Board.

### 2.2. Measures/Outcomes

The primary outcome was having a non-HIV STI diagnosis of interest during an ambulatory office visit. The non-HIV STI diagnosis of interest is a composite outcome measure including chlamydia, gonorrhea, syphilis, HPV, trichomoniasis, chancroid, genital herpes, unspecified non-HIV STI, Reiter’s disease, and granuloma inguinale. We mainly used the Healthcare Cost and Utilization (HCUP)’s Clinical Classifications Software (CCS) and Clinical Classifications Software Refined (CCSR) to identify most of the STI diagnoses (see Table 1) [12,13]. Genital herpes, which is not included in the CCS and CCSR STI categories, was added using diagnosis codes. Since NAMCS recorded three diagnosis codes for each visit from 2005–2013, and five from 2014–2016, only the first three diagnosis fields reported were used in order to maintain consistency throughout the study period. Sensitivity analysis was conducted using all available diagnosis codes.

### 2.3. Statistical Analysis

To calculate reliable estimates with sufficient observations per period, data were combined into three 4-year periods: 2005–2008, 2009–2012, and 2013–2016. This is because NCHS recommended having unweighted frequencies ≥30 or relative standard errors ≤30% to enhance reliability of estimates when using NAMCS data [14]. For individual STI subgroup analyses, we combined diagnoses of gonorrhea (total *N* = 31 unweighted) and syphilis (total *N* = 35 unweighted) due to small sample sizes.

Proc Surveyfreq in SAS was used to obtain survey-weighted estimates of the prevalence rate (per 100,000 visits) of ambulatory care visits that included an STI diagnosis. Multivariable logistic regression was used to examine trends in the proportion of visits related to STIs and identify risk factors associated with a visit with an STI diagnosis compared to those without an STI diagnosis, controlling for age, sex, race, ethnicity, region, insurance type, and HIV status. These variables were selected because prior studies identified them as possible determinants of STIs [15,16]. The Rao–Scott chi-square test was used to compare demographic changes across the three time periods. All statistical analyses were 2-tailed, *p* < 0.05, and conducted using SAS version 9.4 (SAS Institute Inc., Cary, NC, USA).

## 3. Results

From 2005–2016, there were an estimated 19.5 million weighted, office-based ambulatory visits for STIs among patients aged 15–64 years (Table 2). We observed significant increasing trends of non-HIV STI-related visits among individuals who were aged 25–44 (36% to 65%), male (25% to 45%), Hispanic (14% to 20%), and residing in the western U.S. (20% to 28%) from 2005–2008 to 2013–2016 (all *p*-values < 0.001).

### 3.1. Trends of Non-HIV STI-Related Visits

**Figure 1:**
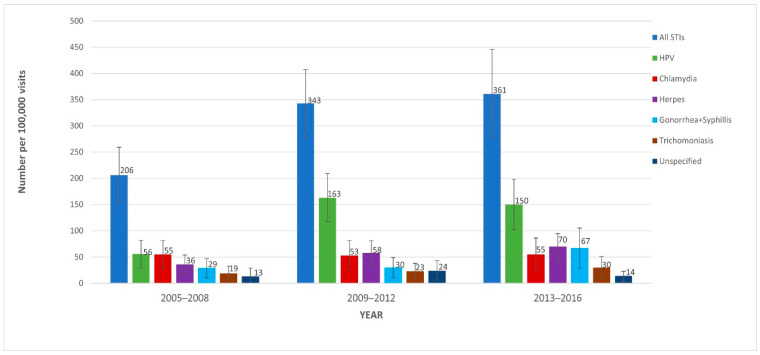
Prevalence of sexually transmitted infection (STI) diagnoses in ambulatory care settings from 2005–2008 to 2013–2016 using data from the National Ambulatory Medical Care Survey (NAMCS). All STIs: chancroid, chlamydia, granuloma inguinale, gonorrhea, herpes, HPV, syphilis, trichomoniasis, unspecified STI, and Reiter’s disease; HPV: human papillomavirus. Note: logistic regression was used to compare the three time periods for all STIs (*p* = 0.003).

Figure 1 displays the trends in non-HIV STI-related office visits from 2005 to 2016. STI-related visits per 100,000 visits to physician offices increased by over 75%, going from 206 (95% CI = 153–259) in 2005–2008, to 343 (95% CI = 279–407) in 2009–2012, and to 361 (95% CI = 277–446) in 2013–2016 (P_trend_ = 0.003). In sensitivity and subgroup analyses, this trend was found to be consistent when using all available diagnosis codes for each time period. HPV-related visits per 100,000 visits were 56 (95% CI = 29–82) in 2005–2008, 163 (95% CI = 117–209) in 2009–2012, and 150 (95% CI = 102–197) in 2013–2016. Of these, females comprised 76.7%, 60.3%, and 52.1% of (HPV-related) visits (See Supplementary Materials Table S1), respectively. Combined syphilis- and gonorrhea-related visits were 29 (95% CI = 11–47), 30 (95% CI = 11–49), and 67 (95% CI = 29–105) visits per 100,000 visits in 2005–2008, 2009–2012, and 2013–2016, respectively. Data on STI-related visits by patients with HIV are shown in Supplementary Materials Table S2.

### 3.2. Factors Associated with STI-Related Visits

Table 3 summarizes factors associated with increased risk of STI diagnosis. Crude odds-ratio estimates are available in Supplementary Materials Table S3. After controlling for these variables, higher odds of an STI-related visit were associated with patients who were younger (aged 15–24: aOR = 4.45; 95% CI = 3.19–6.20 and aged 25–44: aOR = 3.59; 95% CI = 2.71–4.77) vs. aged 45–64, were Black (aOR = 2.41; 95% CI = 1.78–3.25) vs. White, and who had a concomitant HIV infection (aOR = 10.60; 95% CI = 5.50–20.27) vs. without. There was no statistically significant difference between different sexes, regions, or insurance variables.

## 4. Discussion

In this analysis of STIs in ambulatory care settings in a nationally representative sample of U.S. residents, we found an increasing trend in non-HIV STI-related visits from 2005–2016. The prevalence of STI-related office visits increased by over 75% during those years. Younger age and Black race were identified as patient characteristics associated with higher odds of non-HIV STI-related office visits. It is important to note that these groups are among the most affected sub-populations at risk for new HIV infections in the U.S. [17].

This study’s increasing prevalence of STI-related visits aligns with the 2018 CDC Sexually Transmitted Disease (STD) Surveillance reports [3]. It is noteworthy that the CDC only requires reporting of four STIs (chlamydia, gonorrhea, syphilis, and chancroid) to the NNDSS [9]. In contrast, our study used nationally representative data on all STIs directly from physician offices. Results of our sub-analysis on individual STIs in 2013–2016 showed that HPV infections accounted for the highest number of STI-related visits, followed by genital herpes, chlamydia, and trichomoniasis. These results were also consistent with a study by Weinstock et al. [5], which used a modeling approach that estimated HPV and herpes as the top two prevalent STIs in the general U.S. population in 2018. Our study results, which found that HPV, genital herpes, chlamydia, and trichomoniasis comprised 82% of the STIs in 2013–2016 in our sample, are also consistent with work by Kreisel et al., which found that those four STIs comprised 97.6% of all prevalent STIs in the U.S. in 2018 using the National Health and Nutrition Examination Survey (NHANES) data [1]. This difference likely reflects that not all patients with a STI would see a physician due to asymptomatic conditions and thus might not have a corresponding diagnosis recorded in their patient record.

Our results show that the prevalence of STI-related visits in the general population increased across all three time periods. The years 2005–2008 to 2009–2012 had a 62% increase in prevalence, compared to a 6% increase from 2009–2012 to 2013–2016. Notably, our analysis showed very alarming substantial increases in the gonorrhea and syphilis composite from 2009–2012 to 2013–2016. Combined, these STIs had a 123% increase from 2009–2012 to 2013–2016 and were primarily responsible for the overall rise in STIs between 2009–2012 and 2013–2016. It is noteworthy that these two STIs have been linked to HIV [18,19,20] and PrEP use [21]. A mechanism by which syphilis increases HIV transmission is through the disruption of the skin’s lining, enabling the passage of HIV [22]. Prior studies have shown an increase in seminal HIV infection in patients with co-occurring gonorrhea, leading to increased HIV transmission [23]. Furthermore, this is important since some studies have suggested that the increasing STI rates (including syphilis and gonorrhea) may be related to the availability of PrEP, which became available in 2012, and risk compensation amongst PrEP users [7,24,25,26]. Future studies must continue to investigate the association between PrEP use and the risk of STIs.

There was a noticeable decrease in STI-related visits in females; however, the prevalence of such visits was still higher than STI-related visits in males throughout the study. This decrease is possibly associated with the uptake of the HPV vaccine, which in 2006 was initially indicated only for women [27] before an eventual recommendation for males in 2011 by the Advisory Committee on Immunization Practices (ACIP) [28]. HPV-related STI visits increased by 191% from 2005–2008 to 2009–2012, accounting for the increase in overall STI visits between the periods, but then they slightly decreased in 2013–2016. For HPV-related visits, female visits decreased from 76.7% to 52.1% from 2005–2008 to 2013–2016. This may reflect the effectiveness of the HPV vaccine, as studies show that it effectively prevents genital warts, persistent HPV infections, and cervical lesions [29]. An analysis of HPV prevalence using NHANES from 2003–2006 (pre-vaccine era) to 2015–2018 suggested an 86–97% reduction in HPV types 6, 11, 16, and 18 amongst sexually experienced females aged 14–24 who received at least one dose of the HPV vaccine [30]. We considered if changes in HPV screening rates played a factor in our results, however, available data suggest only a slight change over time: 83.93% and 80.78% of women aged 21–65 years were up-to-date with cervical cancer screening in 2005 and 2015, respectively [31].

Patients in the 25–44-year age group accounted for the highest frequency of STI-related diagnoses across all three time periods. Our odds-ratio estimates showed that those aged 15–24 and 25–44 were more likely to have an STI-related visit than 45–64 year-olds. This is consistent with prior studies demonstrating that the rates of risky sexual behaviors escalate during adolescence, reaching a peak in early adulthood [32]. The risky sexual behavior in adolescents has previously been attributed to their underdeveloped decision-making skills [32]. It is fathomable that these patterns of risky sexual behavior may then extend into early adulthood. This result highlights the need for educational campaigns that promote condoms in young adults who may not fully appreciate STI risks. Educating younger patients about these risks (e.g., infertility, cervical cancer, and HIV acquisition) [33,34] is key to tackling STI increases within these populations.

We also noted that Black (vs. White) patients were more likely to have a STI-related office visit. This is consistent with the 2019 STD Surveillance report showing that Black individuals had the highest reported cases for chlamydia, gonorrhea, and syphilis within the 10–34-year age groups [35]. Fergus et al.’s study results suggesting that African Americans “exhibited the highest rate of sexual risk behavior in ninth grade” [32] reveals the need for early intervention efforts to curtail STIs in this population. Budget allocations geared towards health-awareness programs and sex education in middle and high schools in minority communities is essential.

### Limitations

First, due to the frequent asymptomatic nature of STIs, our study may underestimate the total burden of STIs. Hence, our estimate may be more reflective of symptomatic patients or asymptomatic patients screened as of the time of their office visit. Second, there was a small sample size for each STI, so one must be careful in interpreting the results. Third, NAMCS data lack certain variables (e.g., identifiers for MSM or transgender individuals) necessary to completely analyze all potential risk factors for an STI-related visit. PrEP usage was also not included in our analysis due to the very low unweighted frequency. Additionally, while it may be possible that missing or miscoded claims could affect the results of this analysis, it is important to note that coding errors are likely consistent over the three time periods of the study. Lastly, data from Community Health Centers were not included because the CDC has not released recent data. Again, this may cause an underestimation of STI prevalence.

## 5. Conclusions

Using nationally representative survey data in the U.S., STI-related office visits increased by over 75% from 2005 to 2016 in ambulatory care settings. The increase in HPV-related visits largely drove the increase from 2005–2008 to 2009–2012. The increase from 2009–2012 to 2013–2016 is explained by substantial increases in syphilis and gonorrhea. Our findings suggest that adolescents, young adults, and those of Black race were associated with STI-related visits. Further investigation is needed to explore all possible factors associated with STI increases over time. This will enable the design of more specific interventions to mitigate the increasing rates of STIs in order to prevent their complications, financial burdens, and HIV acquisition.

## Figures and Tables

**Table 1:** Table of diagnosis codes for STIs included in the study.

STI	Diagnosis Codes from HCUP’s CCSR and CCS	
	ICD-9-CM	ICD-10-CM
Chlamydia	076x, 078.88, 079.98, 079.88, 099.1, 099.41, 099.5x	A55x, A56x, A71x, A74x
Gonorrhea	098x	A54x
Syphilis	090x–097x	A51x–A53x
HPV	078.11, 795.05, 795.09, 795.15, 795.19, 796.75, 796.79	A63.0, R85.81x, R85.82x, R87.81x, R87.82x
Trichomoniasis	131x	A59x
Chancroid	099.0	A57x
Genital herpes	054.1x	A60x
Unspecified STI	099.9	A64
Reiter’s disease	099.3	M02.3
Granuloma inguinale	099.2	A58

CCS: Clinical Classifications Software; CCSR: Clinical Classifications Software Refined; ICD-9-CM: International Classification of Diseases, Ninth Revision, Clinical Modification; ICD-10-CM: International Classification of Diseases, Tenth Revision, Clinical Modification; HCUP: Healthcare Cost and Utilization; HPV: Human papillomavirus.

**Table 2:** Demographic differences in STI by each time period using data from NAMCS.

Year (*N* = Unweighted # of Visits)	2005–2008 (*N* = 110)	2009–2012 (*N* = 241)	2013–2016 (*N* = 246)	*p*-Value
Weighted number of visits	4,419,914	7,604,358	7,502,703	N/A
	Weighted proportion of visits *N*% (95% CI:)			
Age categories				**<0.01**
15–24 years	35.3 (24.2–46.5)	30.0 (22.0–379)	13.9 (7.7–20.2)	
25–44 years	36.2 (26.0–45.5)	50.9 (43.5–58.3)	65.1 (55.6–74.7)	
45–64 years	28.4 (17.9–38.9)	19.1 (13.2–25.0)	20.9 (12.2–29.6)	
Sex				0.44
Female	74.8 (64.7–84.8)	64.0 (54.6–73.3)	56.0 (44.1–68.0)	
Male	25.2 (15.2–35.3) *	36.0 (26.7–45.4)	44.0 (32.0–55.9)	
Race				**0.03**
White	66.9 (55.6–78.1)	69.6 (60.4–79.3)	71.3 (61.7–80.9)	
Black	27.0 (15.5–38.5)	27.4 (18.0–36.8)	22.1 (13.3–30.8)	
Other	6.1 (0.9–11.4) *	2.8 (0.4–5.1) *	6.7 (1.4–11.9) *	
Ethnicity				**<0.01**
Hispanic	13.6 (6.9–20.2) *	13.6 (7.1–20.2)	19.9 (11.4–28.3)	
Not Hispanic	86.4 (79.8–93.0)	86.4 (79.8–92.9)	80.1 (71.7–88.6)	
Insurance				**<0.01**
Private	68.3 (53.6–83.0)	62.9 (51.8–74.0)	60.3 (48.8–71.8)	
Medicare/Medicaid	17.3 (5.0–29.6)	22.4 (14.2–30.5)	25.0 (14.4–35.6)	
others/missing	14.3 (3.9–24.7)	14.7 (5.1–24.3)	14.7 (6.1–23.3)	
Region				0.37
Northeast	12.8 (5.7–20.0)	15.3 (9.0–21.7)	17.3 (7.9–26.7)	
Midwest	19.7 (8.5–30.9)	13.3 (8.1–18.6)	19.2 (11.3–27.1)	
South	47.5 (33.3–61.7)	54.9 (45.5–64.4)	35.7 (24.6–46.7)	
West	20.0 (10.3–29.6)	16.4 (10.0–228)	27.8 (16.0–39.6)	
HIV diagnosis	1.0 (0.0–3.3) *	3.8 (0.0–8.5) *	3.7 (0.4–7.0) *	0.66

HIV: human immunodeficiency virus. * = data with unweighted frequencies <30 or relative standard error >30 (interpret cautiously). Boldface indicates statistical significance (*p* < 0.05).

**Table 3:** Adjusted odds-ratios of risk factors and STI-related visits using data from NAMCS (*p* = 0.002).

Variables	Adjusted Odds-Ratio (95% CI)
Year of diagnosis (ref: 2005–2008)	
2009–2012	**1.66 (1.20–2.30)**
2013–2016	**1.77 (1.26–2.47)**
Patient age categories (ref: 45–65 years)	
15–24 years	**4.45 (3.19–6.20)**
25–44 years	**3.59 (2.71–4.77)**
Sex (ref: Male)	
Female	0.94 (0.72–1.22)
Race (ref: White)	
Black	**2.41 (1.78–3.25)**
Other	1.08 (0.63–1.84)
Patient ethnicity (ref: Non-Hispanic or Latino)	
Hispanic or Latino	1.31 (0.97–1.78)
Insurance (ref: Medicaid/Medicare)	
Private	0.87 (0.63–1.19)
Others	0.89 (0.53–1.49)
Region (ref: Midwest)	
Northeast	0.93 (0.62–1.38)
South	1.22 (0.90–1.64)
West	1.12 (0.76–1.67)
HIV vs. Non-HIV	**10.6 (5.50–20.27)**

CI: confidence interval; HIV: human immunodeficiency virus. Unweighted number of visits by patients with HIV in 2005–2008, 2009–2012, and 2013–2016 were 1, 8, and 6, respectively; ref: reference. Boldface indicates statistical significance (*p* < 0.05).

## Data Availability

The data presented in this study are openly available with the National Center for Health Statistics at https://www.cdc.gov/nchs/ahcd/datasets_documentation_related.htm (accessed on 20 October 2020).

## Supplementary Materials

The following supporting information can be downloaded at: https://www.mdpi.com/article/10.3390/jcm11010071/s1, Table S1: Table of HPV-related visits by sex; Table S2: Unweighted Number of STI-related office visits by HIV status; Table S3: Crude odds-ratios of risk factors and STI-related visits using data from NAMCS.
