# Supplementary material for: Trends in Sexually Transmitted Infections in United States Ambulatory Care Clinics from 2005–2016
Source: J Clin Med. 2021 Dec 24;11(1):71. doi: 10.3390/jcm11010071 (PMC8745575; doi:10.3390/jcm11010071)
Supplement: Supplementary file 1 [file jcm-11-00071-s001.zip › jcm-1512002-supplementary.pdf]

Table S1. Table of HPV-related visits by sex.

Year of Diagnosis	HPV-Related Visits by Sex *N*% (95% CI)	
	**Male**	**Female**
2005–2008	23.27 (4.54–42.00)	76.73 (58.00–95.46)
2009–2012	39.71 (27.18–52.25)	60.29 (47.75–72.82)
2013–2016	47.87 (31.88–63.87)	52.13 (36.13–68.12)

HPV: Human Papillomavirus.

Table S2. Unweighted Number of STI-related office visits by HIV status.

Year of Diagnosis	STI-Related Visits by HIV Status	
	**HIV**	**Non-HIV**
2005–2008	1	109
2009–2012	8	233
2013–2016	6	240

HIV: Human Immunodeficiency Virus. Note: National Center for Health Statistics recommends reporting unweighted frequencies when they are <30.

Table S3. Crude odds-ratios of risk factors and STI-related visits using data from NAMCS.

Variables	Crude Odds ratio (95% CI)
Year of diagnosis (ref: 2005–2008)	
2009–2012	**1.66 (1.19–2.32)**
2013–2016	**1.75 (1.24–2.49)**
Patient age categories (ref: 45–65 years)	
15–24 years	**4.45 (3.19–6.34)**
25–44 years	**3.63 (2.74–4.84)**
Sex (ref: Male)	
Female	1.05 (0.80–1.39)
Race (ref: White)	
Black	**2.67 (1.98–3.61)**
Other	1.11 (0.66–1.86)
Patient ethnicity (ref: Non-Hispanic or Latino)	
Hispanic or Latino	1.37 (0.99–1.89)
Insurance (ref: Medicaid/Medicare)	
Private	**0.68 (0.49–0.94)**
Others	0.80 (0.48–1.33)
Region (ref: Midwest)	
Northeast	0.94 (0.63–1.40)
South	**1.45 (1.06–1.97)**
West	1.17 (0.79–1.73)
HIV vs. Non-HIV	**14.3 (7.49–27.45)**

STI: sexually transmitted infection; NAMCS: National Ambulatory Medical Care Survey; CI: confidence interval; HIV: human immunodeficiency virus; ref: reference. Boldface indicates statistical significance ($p < 0.05$).
